# Adaptation of a laboratory protocol to quantity microplastics contamination in estuarine waters

**DOI:** 10.1016/j.mex.2019.03.027

**Published:** 2019-04-04

**Authors:** S.M. Rodrigues, C. Marisa R. Almeida, S. Ramos

**Affiliations:** CIIMAR, Interdisciplinary Centre of Marine and Environmental Research University of Porto, Portugal

**Keywords:** Estuarine waters microplastics quantification protocol, Microplastics, Estuarine waters, Contamination, Analytical procedure

## Abstract

One of the most used protocols to extract and quantify MPs is NOAA protocol in aquatic environments. However, there is still no standardized method to extract and quantify MPs in estuarine waters. The aim of this work was to adapt the NOAA protocol to quantify microplastics in estuarine water and provide all the details and changes to improve the efficiency of the method. For that, four types of plastic (PE-LD; PET; PA; PE-HD) were used in artificial samples to test all the steps of the protocol. Several criteria were tested, namely: (i) quantities of H_2_O_2_ used for organic matter degradation; (ii) temperatures of drying samples; and (iii) density separation efficacy. With the proposed modifications, the microplastics extraction were above 90%, regardless the type of plastic, with PE-LD reaching 100% of efficiency. The new adapted protocol that we propose will allow a better efficiency in extraction and quantification of microplastics in samples from estuarine environments.

• Four different types of plastic (PE-LD; PET; PA; PE-HD) were used to test the efficiency of the protocol
• Details as the ideal quantity of H_2_O_2_, temperature and exact quantity of NaCl were tested and defined during the experiments
• Efficiency of the microplastics extraction were above 90%

## Graphical abstract

**Figure:**
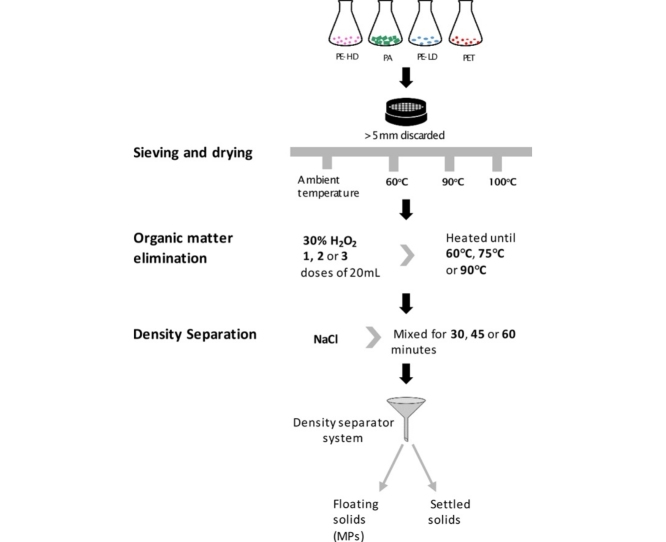

**Specifications Table**

**Table:** 

Subject Area:	*Environmental Science*
More specific subject area:	*NA*
Method name:	*Estuarine waters microplastics quantification protocol*
Name and reference of original method:	*Masura et al.* [1]
Resource availability:	*NA*

## Method details

One of the procedures typically used to analyze microplastics (MPs) in the marine environments is the NOAA protocol [1]. This method is composed by three main phases: sieving and drying (*phase 1*), organic matter elimination or oxidation step (*phase 2*), and density separation (*phase 3*) [1]. However, sampling, separation and quantification of MPs vary widely, being also dependent on the sampling location. The characteristics of estuarine waters differ greatly from seawater samples, mainly in terms of organic matter content that can vary significantly. Furthermore, other studies refer the use of different concentrations of H_2_O_2_ or even other reagents [2,3] as being more effective in degrading all the organic matter present in the sample *(phase 2).* These studies also refer that sodium chloride might not be efficient in the density separation step *(phase 3)* of NOAA protocol, decreasing the protocol’s efficiency and resulting in an underestimation of MPs concentration [4]. All of these factors highlight the need to adapt and establish an efficient methodology specific to estuarine waters to quantify MPs and ensuring an adequate protocol to quantify MPs in the Douro estuary (NW, Portugal) [5].

The NOAA protocol [1] was tested with two types of samples: laboratory samples containing a known type and concentration of MPs; and environmental samples, i.e. estuarine waters. Experimental assays were designed to test: (i) the efficiency of organic matter elimination, (ii) the efficiency of the NOAA protocol in terms of MPs recovery, and (iii) the possible degradation or loss of MPs during the execution of the protocol. The laboratory samples were subjected to all of the three phases of the protocol and environmental samples were subjected to phase 1 and 2, to test the time of drying samples and efficiency of the hot digestion in the elimination of the organic matter.

## MPs preparation

**Fig. 1:**
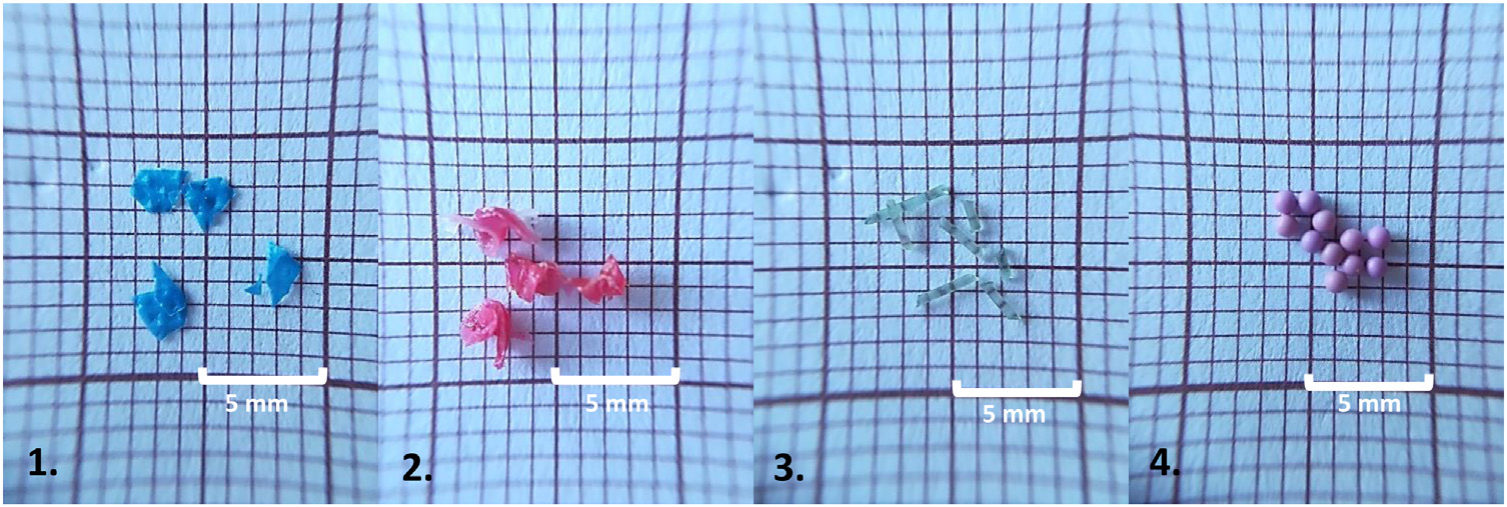
Different types of MPs used: 1. film plastic bags (PE-LD); 2. bottle caps particles (PET); 3. fishing line fibers (PA); 4. microspheres (PE-HD).

The MPs types used in this study were selected from the most common items and types of polymers of marine litter [6], namely: *film plastic bags* (Low-density Polyethylene (PE-LD); specific gravity 0.93 g/cm^3^), *bottle caps particles* (Polyethylene terephthalate (PET); 1.37 g/cm^3^), *fishing line fibers* (polyamide (PA); 1.13 g/cm^3^) and *microspheres* (High-density Polyethylene (PE-HD); 1 mm, 1.00 g/cm^3^) (Fig. 1). Excepting the microspheres, all the remaining types of MPs (e.g. plastics bags, fishing lines) were manually produced and sieved by 5 mm mesh-sieves to discard particles larger than 5 mm. For film plastic bags and bottle caps particles <5 mm were subdivided into 2 size types: 1) between 3 and 5 mm and 2) below 2 mm. Microspheres were commercially acquired (from Cospheric Innovations in Microtechnology) and used as received. An initial mass of 0.5 mg of each type of plastic was defined per sample. For each type of plastic 6 replicates were prepared.

## Contamination

To avoid contaminations during the entire proceeding, all steps were carried out inside a laminar flow cabin to avoid fiber contamination by air. Also, all the equipment used was made of glass and thoroughly rinsed with filtered deionized water before usage. All the deionized water used during the protocol, including the water used in the solutions and the water used to rinse the material was previously subjected to a filtration system, avoiding any type of contamination. Work surfaces were cleaned with 70% ethanol solution and a lab coat and gloves were worn at all times.

### Phase 1 – sieving and drying

**Fig. 2:**
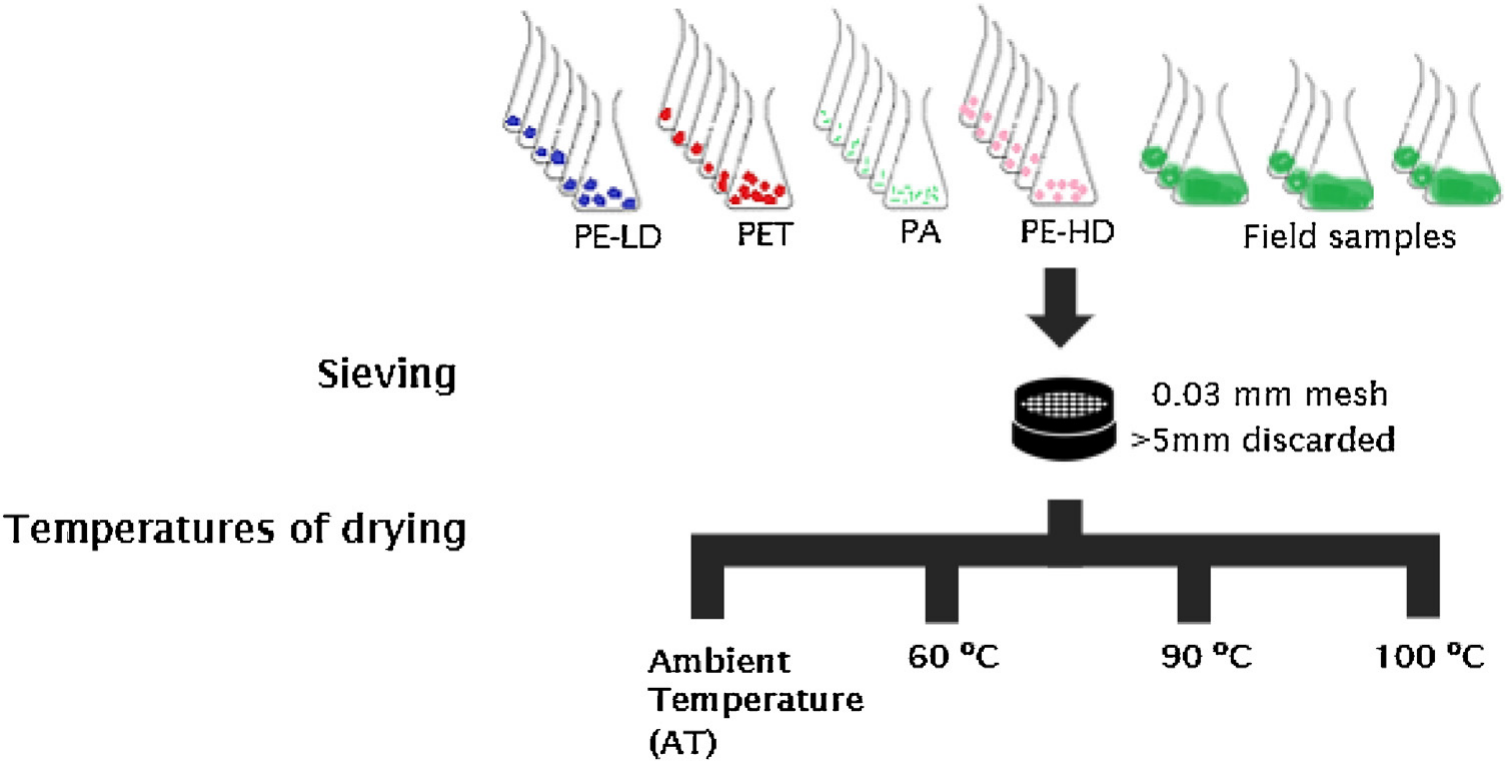
Experimental draw of different temperatures tested in the first phase of protocol, Sieving and drying, in laboratory samples (4 types of MPs) and field samples (3 different stations of the Douro estuary).

The two types of samples were sieved through a 0.03 mm mesh size (thoroughly rinsed with filtered deionized water) and transferred to a new clean and weighed (to the nearest 0.1 mg) glass beaker. Samples were then dried at different temperatures: ambient temperature (AT), 60 °C [7,2,8,9], 90 °C (advised by NOAA), and 100 °C (Fig. 2), to understand if the temperature can decrease the time necessary in *phase 1*. Samples were then weighed (mg) to quantify the total mass of dried matter, including organic and inorganic matter content.

For samples dried at AT it was necessary an average of 48 ± 21 h to completely dry the water. At 60 °C it was still necessary more than one day (31 ± 14 h) until samples were completely dried. Thus, these two temperatures led to an even more time-consuming protocol. However, when we used 90 °C [10] the number of hours needed to dry the samples was significantly lower than at 60 °C or AT (ANOVA F = 15.25 p ≤ 0.01). PE-HD MPs need 4 h to dried, PE-LD and PA completely dried after 6 h, and PET needs 8 h. For field samples 10 ± 3 h were necessary to dry the samples. At 100 °C or with temperatures above, MPs can deform, i.e. altering their initial size and shape. Thus, we consider that 90 °C overnight is the temperature and time more appropriate to dry estuarine water samples in *phase 1*.

### Phase 2 – organic matter elimination

**Fig. 3:**
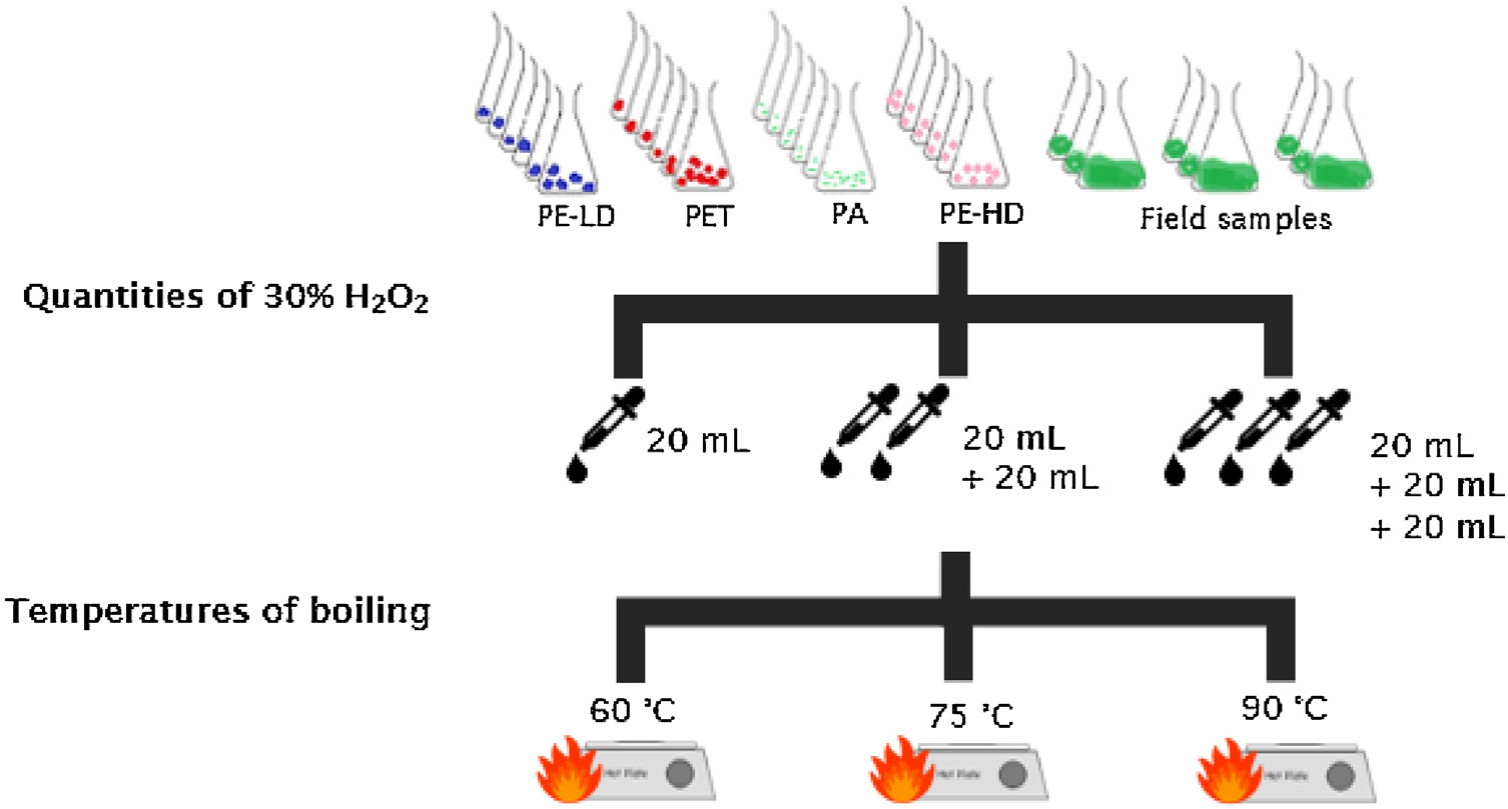
Experimental draw of the quantity of 30% H_2_O_2_ and temperature of boiling tested in the second phase of protocol, organic matter elimination, in laboratory samples (4 types of MPs) and field samples (3 different stations of the Douro estuary).

In the second phase, a mixture of a 0.05 M Fe(II) solution (7.5 g of FeSO_4_.7H_2_O (from SIGMA) in 500 mL of water and 3 mL of concentrated sulfuric acid (from SIGMA-ALDRICH)) with a 30% H_2_O_2_ solution (from ACROS ORGANICS) was used. In our study, the laboratory samples were used to test if the hot digestion destroy/modify the MPs; and environmental samples were used to assess the efficiency of NOAA protocol to eliminate organic matter of samples. Therefore, 20 mL of solution was added to each beaker, being heated until 75 °C (as recommended in NOAA). Higher and lower temperatures (95 °C and 60 °C) were also tested (Fig. 3). The NOAA protocol advises the use of 20 mL doses of 30% H_2_O_2_ solution as many times as needed to ensure that all organic matter, except plastic, is dissolved. In our study, one, two, and three doses of 20 mL of 30% H_2_O_2_ solution were added to 1) assess if different quantities of H_2_O_2_ affected characteristics of the MPs, in laboratory samples and 2) analyze if the temperature and a higher number of doses of 30% H_2_O_2_ would help in the degradation of the organic matter, in field samples. Samples were heated until they boiled, reaching the degradation point, being afterwards kept in the hotplate for 30 min (as advised by NOAA). After, field samples were dried again at 90 °C and weighted to quantify the inorganic content and thus estimate the degradation of organic matter content by the protocol.

During the elimination of organic matter phase, it is crucial that the samples reach the boiling point, since only in that point the degradation reaction happens. To induce the boiling point, the temperature adopted by NOAA protocol, 75 °C, appeared to be the ideal temperature. Our tests showed that temperatures below 75 °C inhibited the reaction, leading to minimum or inexistent organic matter degradation. Above 75 °C the solution boiled too violently leading to loss of material. One limitation of the NOAA protocol was found in this step. The NOAA protocol indicates to use doses of 20 mL of 30% H_2_O_2_ solution as many times as needed to ensure the dissolution of the organic matter present in the sample. However, it was observed that one dose of H_2_O_2_ solution was only effective when the amount of organic matter was minimal (Table 1). For higher amounts of organic matter, a second dose (20 mL + 20 mL) of H_2_O_2_ solution was necessary, being the majority of the organic matter only degraded with two doses (20 mL + 20 mL) of H_2_O_2_. The percentage of organic matter degraded was significantly higher when two doses (20 mL + 20 mL) of H_2_O_2_ were added (ANOVA F = 80.95, p ≤ 0.01) (Table 1). But, in some cases there was still organic matter present in solution. However, adding a third dose of H_2_O_2_ solution was useless and ineffective, as it resulted in the dilution of the reagents, leading to a weak reaction and, consequently, to inefficient degradation of the remaining organic matter. Furthermore, a third dose of H_2_O_2_ leads to changes in size or color in some types of MPs tested (Table 1).

**Table 1:** Results of the organic matter elimination testing different quantities of 30% H_2_O_2_ in laboratory and field samples. In laboratory samples were analyzed the mean (±standard deviation) percentage of degradation, loss, or alteration of MPs and in field samples the mean (±standard deviation) percentage of organic matter elimination.

Organic matter elimination		Quantity of H_2_O_2_ solution		
		20 mL	20 mL + 20 mL	20 mL + 20 mL + 20 mL
Percentage of alterations in MPs	Film plastic bags (PE-LD)	Without changes	Without changes	25.0% ± 0.4 changed in size (fragmentation)
	Bottle caps particles (PET)	Without changes	Without changes	Without changes
	Fishing line fibers (PA)	Without changes	Without changes	100% changed in color (to brownish color)
	Microspheres (PE-HD)	Without changes	Without changes	33.0% ± 0.5 changed in size (fragmentation)
Percentage of organic matter eliminated	Field samples	60.0% ± 0.2	98.00% ± 0.03	0%

### Phase 3 – density separation

**Fig. 4:**
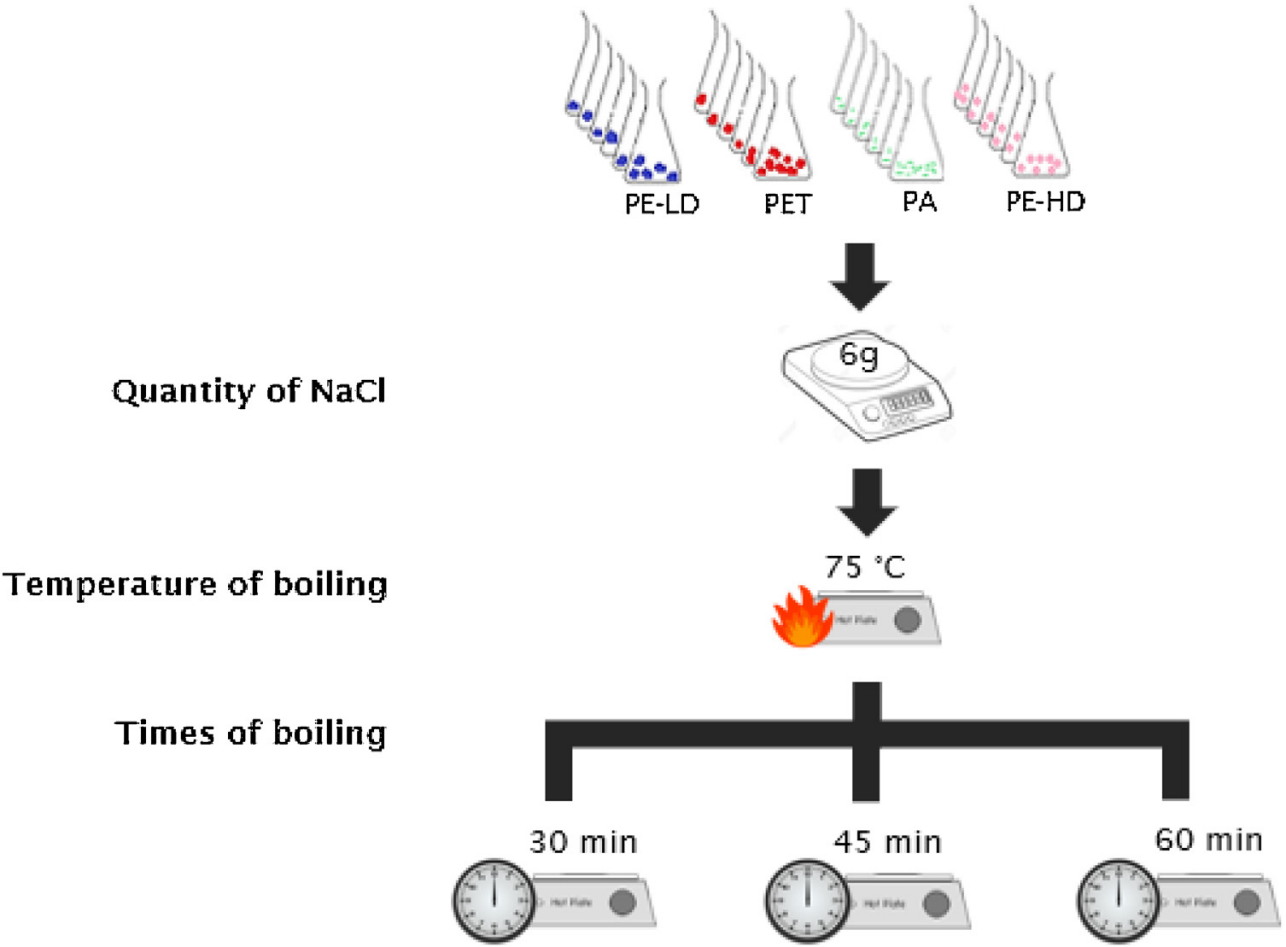
Experimental draw of the quantity of NaCl, temperature and time of boiling tested in the third phase of protocol, density separation, in laboratory samples (4 types of MPs).

To make all MPs float, high density saturated solutions are normally added to the samples. Following to NOAA protocol, in the laboratory samples, a fully-saturated salt solution was prepared by dissolving ˜6 g of NaCl (from SIGMA-ALDRICH) per 20 mL of sample solution. To improve the dissolution, the mixture is then heated to 75 °C and mixed with a stirring magnet for 30 min. In the present study, additional periods of 45 min and 1 h of heating and stirring were also tested (Fig. 4). Afterward, the solution was transferred to the density separator, covered with aluminum foil and left to precipitate overnight. In the following day, the settled solids were collected to visually evaluate if any MP was trapped in the bottom of the density separator and therefore unable to float, or had a higher density leading to their precipitation. The floating solids were drained through a 0.03 mm mesh to guarantee that all the plastics particles were collected. The density separator was rinsed several times with deionized water to transfer all the solids to the mesh. The mesh with the MPs was then closed in a petri dish and dried in an oven at 90 °C.

Afterward, meshes were weighted and then all the solids were collected, weighted, and visually inspected under a stereomicroscope (Nikon SMZ800) to quantify the original MPs used, and to look for signs of MPs deterioration. Each type of MP was, at the beginning of the experiment, characterized by color and shape to assess if they were altered during the protocol procedures. The percentage of MP recovered was calculated.

According to the NOAA protocol, the exact quantity of NaCl is not important, referring around 6 g. Our tests showed that this quantity is not easily dissolved, and we observed that when the quantity of NaCl was slightly above 6 g (in our study we used 6.3 g), it was not possible to achieve full dissolution of NaCl (Table 2). Thus, when the samples were placed in the density separator, the NaCl precipitated, trapping part of the MPs, and accumulated on the bottom of the system, blocking the flow of the liquid. If the quantity of NaCl added was a little lower than 6 g (in our study we used 5.7 g), all the NaCl was dissolved but a percentage of the MPs did not float (Table 2). Therefore, we found that for total NaCl dissolution it was necessary to weight precisely 6 g of NaCl per 20 mL of solution and heat the solution for 30 min while using a stirring magnet. The stirring magnet used needs to be high temperature resistant to prevent any deterioration and contamination during its use. Additionally, the stirring manet needs to be rinsed with filtered deionized water: i) before use, to prevent any exterior contamination; ii) before being removed from the glass beaker and carefully examined to unsure it has no particles from the sample attached. This procedure allowed all the microplastics to float. So, this new procedure was added to our proposed protocol. Also, our study showed that increase time did not improve NaCl dissolution, 30 min being selected as the time to heat the samples.

**Table 2:** Results of the density separation testing different quantities of NaCl in laboratory samples. In the laboratory samples was analyzed the mean (±standard deviation.) percentage of dissolution of NaCl and the mean (±standard deviation) percentage of MPs that float in the density * – represents samples where was impossible determine the percentage of MPs that float due the accumulation of NaCl in the bottom of the system trapping part of the MPs and blocking the flow of the liquid.

	Type of MPs	Quantity of NaCl			Type of MPs	Quantity of NaCl	
Dissolution of NaCl	PE-LD	5.7	100%	Floatability of MPs	PE-LD	5.7	90.20% ± 0.05
		6.0	99.25% ± 0.03			6.0	98.18% ± 0.08
		6.3	86.33% ± 0.04			6.3	*
	PET	5.7	100%		PET	5.7	84.11% ± 0.05
		6.0	99.33% ± 0.01			6.0	95.33% ± 0.1
		6.3	87.30% ± 0.05			6.3	*
	PA	5.7	100%		PA	5.7	87.23% ± 0.06
		6.0	100% ± 0.01			6.0	93.33% ± 0.05
		6.3	90.43% ± 0.06			6.3	*
	PE-HD	5.7	100%		PE-HD	5.7	100%
		6.0	100% ± 0.01			6.0	100% ± 0.02
		6.3	85.22% 0.04			6.3	*

**Fig. 5:**
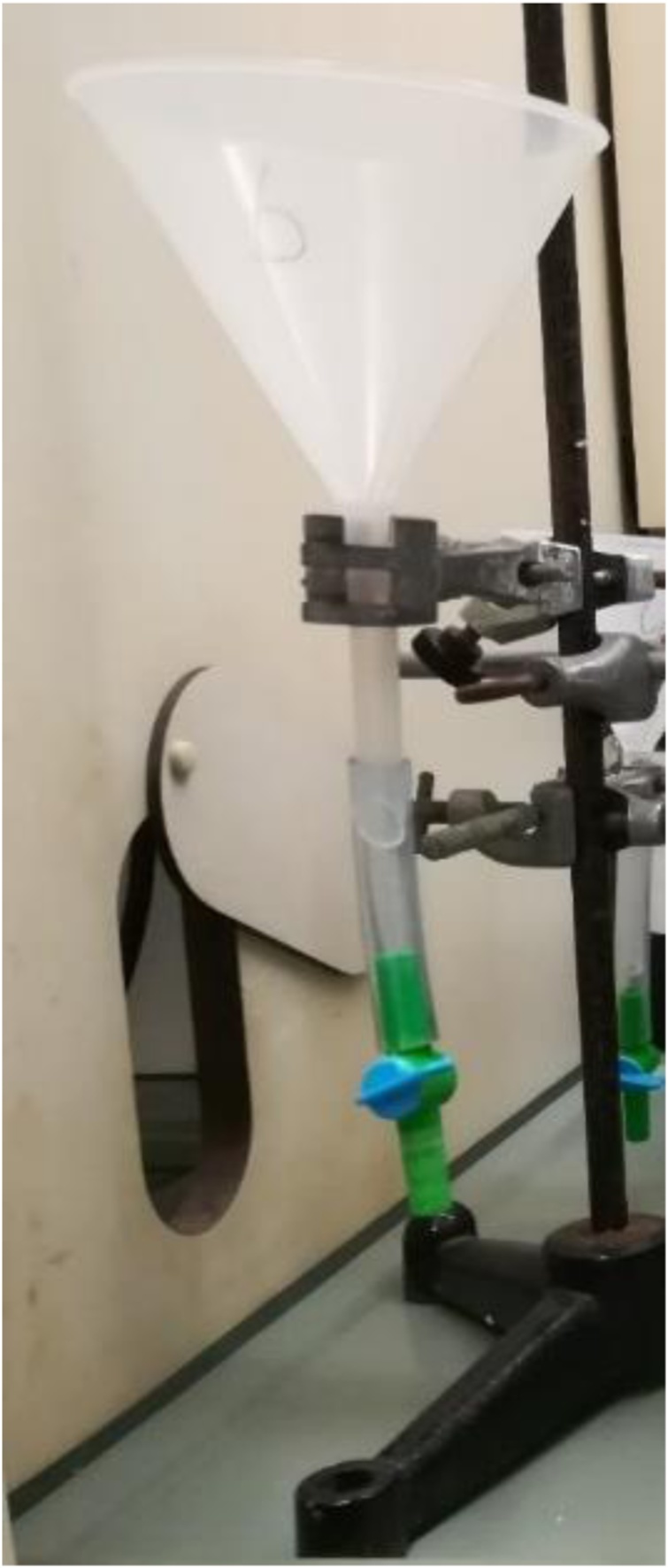
Density separator system used in this study. The system consisted in a glass funnel fitted with a latex tube on the bottom of the stem and a faucet to control de liquid flow.

**Fig. 6:**
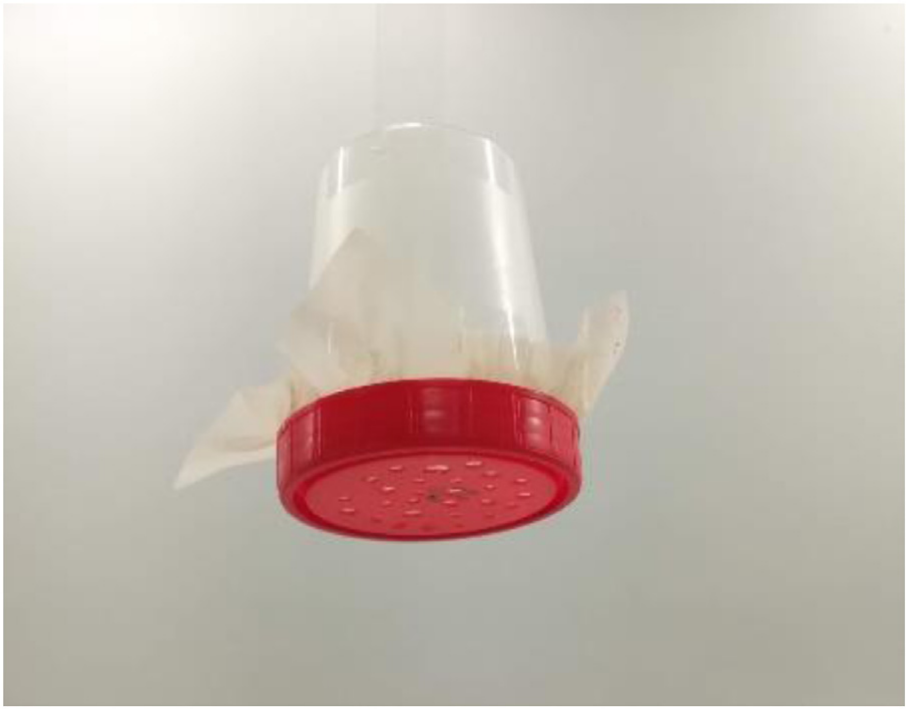
System created and used in this work to wash the mesh with the MPs; consists in an open flask and holes in the lid, the mesh is immobilized between the flask and the lid.

Another issue of the NOAA protocol that required improvements was the step of rinsing the beaker to transfer the sample to the density separator. The protocol advised to use deionized water to rinse particles from the beaker walls, but this step decreased the density of the separation liquid and resulted in sinking particles that had already been afloat. So, to avoid this, the minimum amount of deionized water was used whenever needed. In other studies, the deionized water was substituted with the same separation liquid for rinsing [10]. In relation to the density separator (Fig. 5) we also detected some concerns, and some components were changed to improve the system efficiency. It was observed that the latex tube at the bottom of the separator degraded very quickly, due to the contact with the oxidizing solution, requiring often replacements by a new tube or alternatively the use of a tube made from glass. The NOAA protocol advises on the use of a pinch clamp on the latex tube. However, in this study we opted to use a faucet, becoming easier to control the flow of the solution, discarding it whenever necessary. We noticed that this filtration procedure led to the accumulation of a high amount of NaCl on MPs and on the mesh itself used to collect the floating MPs, which could affect the final weight of the MPs. Thus, we found necessary to include a thorough wash of the mesh and particles to eliminate excesses of NaCl. To solve this issue, we created a system with an open flask to immobilize the mesh (Fig. 6), allowing to immobilize the filter cloth and wash it several times with running deionized water. The filter cloth was then placed in another flask with deionized water in the bottom and covered with aluminum foil and leave to rest, ensuring that all the remaining NaCl was dissolved in the water. All these steps are detailed in our proposed protocol (please see the supplementary material).

**Fig. 7:**
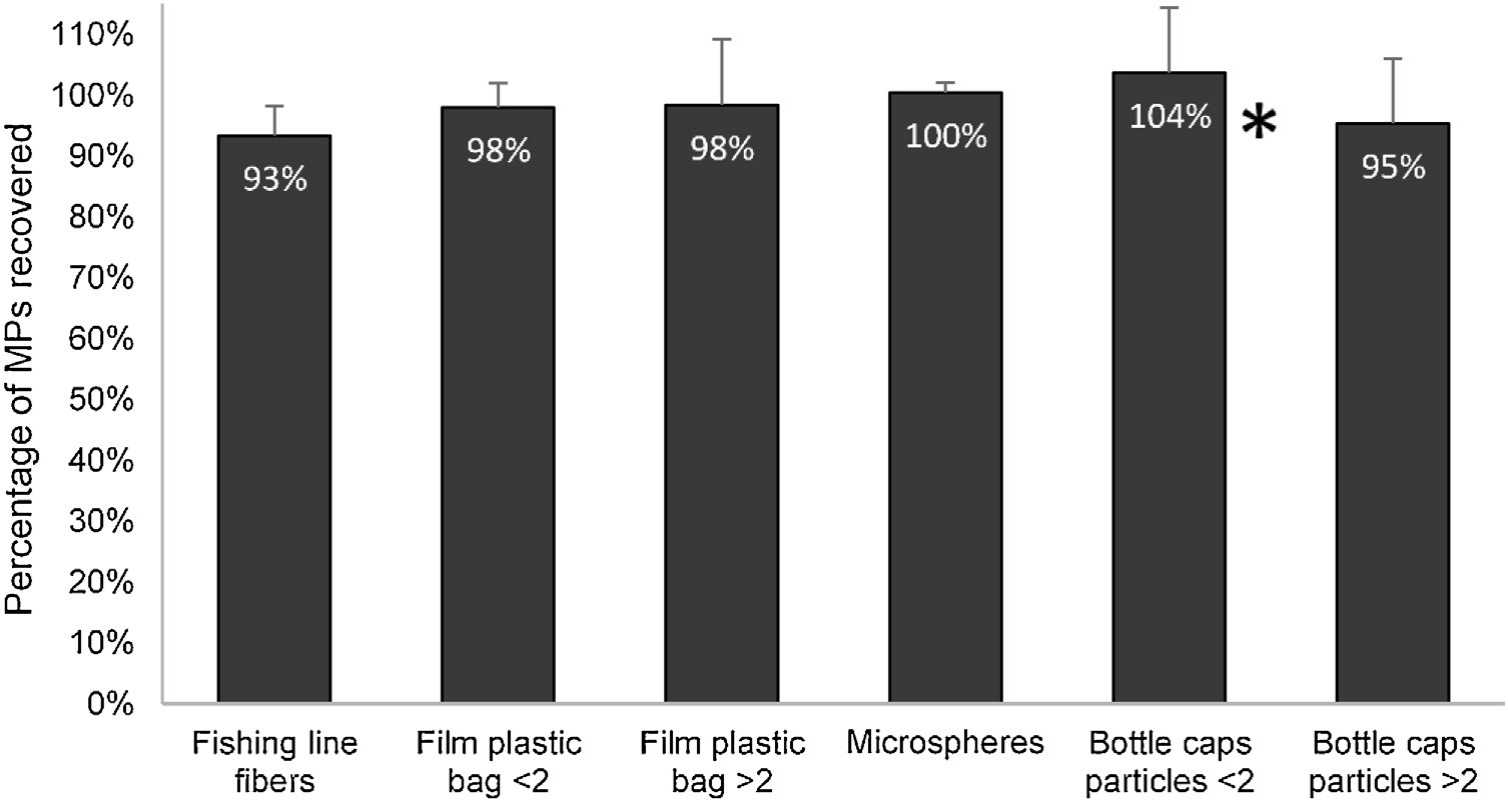
Percentage of microplastics recovered from the top of the density separator system, for each type of plastic subjected to the protocol. Error bars represent the standard deviation associated (n = 6). * – Bottle caps particles <2 had a percentage above 100%, possibly explained by accumulation of NaCl on the rugged parts of this type of MPs.

The majority of MPs were recovered from the top of the density separator, regardless the type of plastic tested. Although the settled solids were always collected and visually inspected, none MPs were found in the bottom of the density separator system or in the settled solids with our adapted protocol. The recovery values were all above 90%, (Fig. 7). The recovery of the PE-HD (microspheres) was the highest, with 100% of recovery. The lowest recovery percentage was observed for PA (fishing line fibers) with only 93% of recovery, what may be explained by potential loss of this type of plastic with the protocol treatments, namely during the organic matter elimination phase. In fact, after phase 2, we noticed that fishing line fibers were visually altered from their transparent color to more brownish colors. So, it is important to consider that the NOAA protocol might underestimate the number of this type of fibers. In the case of PE-LD (film plastic bag) MPs there were no differences in the percentage of recovery between MPs <2 mm and MPs >2 mm. In the case of PET (bottle caps particles) there was a slight difference in the efficiency of recovery (104% for PET < 2 mm and 95% for PET > 2 mm) between MPs sizes, but without significant differences between the two size classes (ANOVA F = 1.83 p ≥ 0.21). In the case of PET MPs (bottle caps particles) <2 mm, the efficiency of recovery was slightly above 100%. One of the greatest issues during the use of the NOAA protocol was the accumulation of NaCl around MPs in the separation step, mainly in the bottle caps particles. It is thus possible that some NaCl precipitated and accumulated on the rugged parts of these particles leading to an increase of the final weight. This fact highlights the need for a thorough washing of the particles after the separation step, that can be more difficult for smaller particles.

Overall, the recovered MPs masses did not show significant differences between the types of MPs (ANOVA F = 1.15 p ≥ 0.36). These results revealed that no matter the type of MPs, the modified protocol established in our study had a similar efficiency for high and low-density Polyethylene, Polyethylene terephthalate and polyamide.
