# Supplementary material for: Adaptation of a laboratory protocol to quantity microplastics contamination in estuarine waters
Source: MethodsX. 2019 Apr 4;6:740–9. doi: 10.1016/j.mex.2019.03.027 (PMC6462778; doi:10.1016/j.mex.2019.03.027)
Supplement: Supplementary file 1 [file mmc1.docx]

**Supplementary material**

Protocol to quantify microplastics in estuarine waters detailing all laboratory procedures updated from NOAA (Masura et al., 2015). To avoid contaminations during the entire proceeding, all steps must be carried out inside a laminar flow cabin to avoid fiber contamination by air. Also, all the equipment that will be used during the protocol must be made of glass and thoroughly rinse with filtered deionized water before usage. All the deionized water used during the protocol, including the water used in the solutions and the water used to rinse the material must be previously subjected to a filtration system, avoiding any type of contamination. Work surfaces must be cleaned with 70% ethanol solution and a lab coat and gloves must be worn at all times.

**1º day**

1. Pass the water sample through a filter cloth (mesh <0.03 mm previously rinsed with deionized water and completely dried)
2. Rinse the water containing recipient thoroughly with a squirt bottle filled with deionized water to transfer all residual solids to the filter cloth
3. Rinse the cloth thoroughly with deionized water. Ensure all material has been well washed, drained, and sorted
4. Weigh a clean and dry 500 ml beaker to the nearest 0.1 mg
5. Transfer solids collected in the filter cloth into the beaker using a spatula and minimal rinsing with a squirt bottle containing deionized water
6. Ensure all solids are transferred into the beaker
7. Place beaker drying in oven at 90 °C overnight

**2º day**

1. Weigh the beaker with the dried sample to stable weight, to ensure dryness
2. Turn on the hotplate to 75 °C
3. Add 20 mL of aqueous 0.05 M Fe(II) solution to the beaker containing the fraction of collected solids and then 20 mL of 30% H_2_O_2_ solution
4. Add a stir bar to the beaker and place them in the hotplate inside the fume hood
5. Heat to 75 °C for 30 minutes until gas bubbles are observed at the surface. If the reaction is too violent, remove the beaker from the hotplate until boiling subsides. If reaction appears to have the potential to overflow the beaker, add deionized water to slow the reaction.
6. When the reaction stops (no boiling) add another 20 mL of 30% H_2_O_2_ solution and heat to 75 °C letting it react as describe before (step before).
7. Add precisely 6 g of salt (NaCl) per 20 mL of sample to increase the density of the aqueous solution (ca. 5 M NaCl) (18 g of NaCl if you add 20 mL Fe(II) solution **+** 20 mL 30% H_2_O_2_ solution + 20 mL 30% H_2_O_2_ solution)
8. Heat mixture to 75 °C for 30 minutes or until the salt dissolves
9. Transfer the saturated solution to the density separator
10. Rinse the saturated solution beaker with deionized water to transfer all remaining solids to the density separator. Use the minimum amount of deionized water as possible to avoid diluting the saturated solution
11. Cover loosely with aluminum foil
12. Allow microplastics to float overnight

**3º day**

1. Visually inspect settled solids for any microplastics. If any is found try to gently hit the separator, making sure all microplastics are floating above
2. Drain settled solids from the separator bottom and discard
3. Weigh a clean and dry filter cloth (mesh <0.03 mm previously rinsed with deionized water and completely dried) to the nearest 0.1 mg
4. Collect floating solids by passing the solution through the filter cloth immobilized in an open flask
5. Rinse the density separator several times with deionized water to transfer all solids to the filter cloth
6. Wash the filter cloth several times with running deionized water and then place the system with the filter cloth in a flask (covered with aluminum foil) with deionized water in the bottom for 5 minutes
7. Place the filter cloth in a petri dish and cover loosely with aluminum foil
8. Allow to dry for 24 hours at 90 °C

**4º day**

1. Weigh the filter cloth with the sample dried to the nearest 0.1 mg
2. Collect all the remain material and microplastics from the filter cloth and weight them
3. Under a dissecting microscope at 40X magnification, use forceps to collect identifiable microplastics from the 0.03 mm mesh and transfer them to a tared vial.
4. Weight only distribution the microplastics
